# Union Generates Success

**DOI:** 10.21470/1678-9741-2017-0063

**Published:** 2017

**Authors:** Domingo M. Braile

**Affiliations:** 1 BJCVS

## Entrepreneurship and Cardiovascular Surgery

In the last weeks, the Brazilian Journal of Cardiovascular Surgery (BJCVS) Blog published three articles concerning the intersection of cardiovascular surgery and entrepreneurship. One of the articles brings the international case of the Indian cardiovascular surgeon Dr. Devi Shetty, who created a health management model that revolutionized Indian reality. A second article dissects a Brazilian case, the startup of the cardiovascular surgeon Dr. Marcus Vinicius Gimenes, named "Consulta do Bem", which seeks to facilitate access to private health care by making use of shared economic model. Finally, the last article discuss the importance of leadership for cardiovascular surgeons and how the concept of leadership changed since the last decades.

I myself, as a cardiovascular surgeon, was compelled to be an entrepreneur, to produce cardiac pericardial valves, not available in the country at the beginning of our specialty. So, I deeply believe that entrepreneurship must be instigated in our young students, residents, and surgeons. Entrepreneurship is the key to progress and, if we want cardiovascular surgery keeps advancing and being known for its innovative nature, we must ensure that the new generations are stimulated and free to create new things.

Another believe I have is that innovation must come from the Universities, in close cooperation with the medical devices Industry. The University is the ideal place which brings together the young, smart and dedicated people, the knowledge accessibility and the resources to develop and test new concepts, to be produced and distributed by the Industries. It was not a coincidence that, within university surgeons, as Prof. Euryclides of Jesus Zerbini and Prof. Adib Jatene, was developed a mindset that we should, create and manufacture many of the equipment for cardiovascular surgery in Brazil. An interesting tool recently created, and which relates to what I mentioned before, is the Brazilian Index of Entrepreneurial Universities. It is a ranking based on how the Universities in Brazil stimulate innovation and entrepreneurship and can be accessed at http://bit.ly/indexofentrepreneurialuniversities. After you check the Index, I invite you to inquire yourself with the following questions: How does my University scores in the ranking? What can I do to improve this score?

With these thoughts, I urge all my surgeons colleagues to learn more about entrepreneurship, develop your ideas and stimulate your fellows to do the same. Only then, we will bring back the innovative spirit which for long characterized the field of cardiovascular surgery.

## 44^th^ Brazilian Congress of Cardiovascular Surgery

From April 20 to 22, the 44^th^ Brazilian Congress of Cardiovascular Surgery will take place at Riocentro Exhibition & Convention Center in Rio de Janeiro, RJ.

The theme this year is "High Technology: Acquiring Knowledge to employ it better".

At the same time, the 7^th^ Symposium of Nursing in Cardiovascular Surgery, the 7^th^ Symposium of Physiotherapy in Cardiovascular Surgery, the 6^th^ Academic Congress on Cardiovascular Surgery, the 35^th^ Brazilian Congress on Cardiopulmonary Circulation, the Meeting of Female Surgeons and the Meeting of Residents, completes the program.

It's scheduled 3 opening lectures by 3 worldwide recognized leading experts. The subjects are the understanding of mitral regurgitation pathophysiology after repair or replacement, the standard of surgical management in coronary artery bypass grafting (CABG) and the role of surgery in treatment of cardiac failure of ischemic etiology are 3 issues of interest to surgeons today.

The presence of 7 several international speakers are: Hartzell Schaff, Mayo Clinic (USA); John D. Puskas, Mount Sinai (USA); Eric Velasquez, Duke University (USA); Andreas Martens, Hannover Medical School (GER); Michael Madani, University of California San Diego (USA); Emir Kabil, Centre for Heart Tuzla (BIH) and Vlassis Ninios, Interventional Cardiologist, St. Luke's Hospital (Greece) which guests consolidate the growth and fundamental integration of SBCCV with international Societies and values the essential exchange of knowledge. In addition, participants will enjoy moments with colleagues and family as well as leisure time visiting the beautiful capital of Rio de Janeiro.

The Scientific Methodological course that will happen on April 20^th^ and TAVI, TEVAR, HPTEC - chronic thromboembolic pulmonary hypertension and intraoperative echocardiogram course that will happen on April, 22^rd^ are the news of the 44^th^ SBCCV Congress, bringing great professionals to share their wisdom.

I congratulate the Board of the SBCCV, the Organizing Committee; coordinated by Dr. Henrique Murad, on the effort made for this meeting to become interesting for all participants of the Congress.

This year the free themes will have an award differently. The SBCCV has implemented several educational projects, including the free themes of the congress, with the following awards: 1^st^ place - Airline tickets and international congress registration (AATS, STS or EACTS). 2^nd^ place - Arline tickets and registration for the next SBCCV Congress and 3^rd^ place it is a registration for the next SBCCV Congress. These projects aim to contribute as encouragement scientific production for each specialty.

I proudly announce that in this issue we are publishing the first article "The Brazilian Registry of Adult Patient Undergoing Cardiovascular Surgery, the BYPASS Project: Results of the First 1,722 Patients" (page 71), which is the beginning to come true an old dream of the SBCCV that it will allow the access of safety database about cardiovascular surgery in the country. The BYPASS, will also be introduced by Dr. Walter Gomes at clinical studies table, that impacts surgical procedures on Friday.

BJCVS will have a stand working together with SBCCV, that it will host every participant during the meeting.

During the Welcome Cocktail event, there will be the prelaunch of the book "Cardiovascular Surgery - A Clinical Casebook" of academic league of SBCCV.

## Meeting of the Editorial Board of BJCVS with Associate Editors and Members of the Editorial Board.

On April 21^st^, from 1pm to 2pm, in room 211, there will be a meeting of the Editorial Board of the BJCVS with Associate Editors and Editorial Board members, also open to all members. We will address very strongly the issues like financial, scientific, ScholarOne, ABEC, Plagiarism, CrossRef, DOI, ORCID, IF, planning 2017/2018 and it will be of great importance to have all suggestions and criticisms in this, so that we can improve the Journal and make it in accordance with the requirements of all Databases and aspiration of our readers around the globe. I count on the participation and suggestions from everyone.

My warmest regards,

**Domingo M. Braile** ^1^Editor-in-Chief - BJCVS

